# Bitter Taste Signalling via TAS2R43 Enhances Temozolomide Efficacy in Glioblastoma Cells

**DOI:** 10.3390/ijms27073262

**Published:** 2026-04-03

**Authors:** Ana R. Costa, Ana C. Duarte, Isabel Gonçalves, Robert Preissner, José F. Cascalheira, Helena Marcelino, Cecília R. A. Santos

**Affiliations:** 1 RISE-Health, Department of Medical Sciences, Faculty of Health Sciences, University of Beira Interior, 6200-506 Covilhã, Portugal; acduarte@fcsaude.ubi.pt (A.C.D.); igoncalves@fcsaude.ubi.pt (I.G.); jfcascalheira@ubi.pt (J.F.C.); htmarcelino@fcsaude.ubi.pt (H.M.); 2 Institute of Physiology and Science-IT, Charité-Universitätsmedizin Berlin, Corporate Member of Freie Universität Berlin, Humboldt-Universität zu Berlin, and Berlin Institute of Health, Philippstrasse 12, 10115 Berlin, Germany; robert.preissner@charite.de; 3 RISE-Health, Department of Chemistry, Faculty of Sciences, University of Beira Interior, 6200-506 Covilhã, Portugal

**Keywords:** glioblastoma, temozolomide, bitter taste receptors, taste signalling, TAS2R43

## Abstract

Bitter taste receptors (TAS2Rs) are increasingly recognised as extraoral chemosensors that modulate diverse biological processes, including cancer cell behaviour and drug responsiveness. Many TAS2R ligands correspond to therapeutic compounds; however, their contribution to the response of brain tumours to chemotherapy remains unexplored. Here, we investigated whether the bitter taste signalling pathway is modulated by temozolomide (TMZ), the standard chemotherapeutic agent for glioblastoma, with an impact on treatment efficacy in glioblastoma cells. We show that TMZ elicits intracellular Ca^2+^ responses compatible with activation of G-protein-coupled receptor signalling and induces anti-proliferative and pro-apoptotic effects in multiple human glioblastoma cell lines. Pharmacological inhibition of bitter taste receptors, as well as genetic silencing of the taste transduction G protein GNAT3, significantly attenuated TMZ-induced cytotoxicity, suggesting that bitter taste signalling is involved in this process. In silico ligand prediction combined with receptor expression profiling identified TAS2R43 as a candidate modulator of these effects, and TAS2R43 knockdown markedly reduced TMZ-induced loss of cell viability and apoptosis. Moreover, TMZ enhanced intracellular accumulation of the ABC transporter substrate doxorubicin, suggesting modulation of multidrug efflux mechanisms. Collectively, our findings identify TAS2R43 as a potential biomarker that warrants further validation to improve responses to TMZ and other ABC transporter-limited anticancer drugs.

## 1. Introduction

Apart from sensing bitter compounds in the oral cavity, bitter taste receptors (TAS2Rs) have also been described in several extraoral tissues, such as the gastrointestinal tract, airway epithelium, heart, testis, and the brain and its barrier interfaces [1,2,3,4]. The human TAS2R family comprises 26 receptors—members of the G protein-coupled receptor (GPCR) superfamily—that together form a broadly tuned bitter chemosensory system [5,6]. Experimental and clinical evidence indicate that TAS2Rs and their ligands contribute to cancer progression and metastasis by regulating key processes such as tumorigenesis, proliferation, migration, invasion, and oncogenic signalling pathways [7,8]. Current evidence suggests predominantly anti-tumorigenic roles for TAS2Rs in several cancers, including improved survival and reduced proliferation and migration in several solid tumours, such as neuroblastoma, breast, and gastrointestinal tumours [9,10,11]. However, whether TAS2R signalling influences the response of highly chemoresistant tumours such as glioblastoma to standard-of-care drugs remains unknown. Temozolomide (TMZ) is an alkylating agent widely used for the treatment of glioblastoma and other neoplasms of the central nervous system (CNS) [12], known for its ability to methylate DNA, resulting in DNA damage and induction of tumour cell death by apoptosis [13]. However, its efficacy is limited because some tumour cells can repair TMZ-induced DNA damage through the action of the O6-methylguanine DNA methyltransferase gene [14], leading to relapse and chemo- and radioresistant cancer stem-like cells, and because these are highly infiltrative tumours [15,16,17]. Additionally, the blood–brain barrier, the blood–cerebrospinal fluid barrier, as well as brain cancer cells overexpress multidrug efflux transporters such as the ATP-binding cassette transporters ABCB1, ABCC1, and ABCG2, which actively extrude several CNS-targeting medicines [18,19,20,21,22], limiting therapeutic concentrations at their target cells and contributing to chemoresistance [23]. Previous work from our group and others reported expression of multiple TAS2Rs in glioblastoma-associated tissues and cell lines, suggesting that a broad subset of the 26 human TAS2Rs may be present in this tumour context [6,24,25]. Among these, TAS2R43 is of particular interest because it is highly expressed in head and neck squamous cell carcinoma cell lines, and it recognises several dietary and drug-like bitter ligands that have been used as co-adjuvants of chemotherapeutics, such as epigallocatechin gallate and other polyphenols [26,27]. Given that TMZ is predicted to be a bitter-tasting compound, we hypothesised that it might bind TAS2Rs and investigated the functional readout of this interaction. We first analysed whether TMZ effects depend on the bitter taste signalling pathway in glioblastoma cell lines and found that the presence of TAS2R43 is important for its anticancer efficacy in these cells.

## 2. Results

### 2.1. TMZ Elicits Intracellular Ca^2+^ Responses in Glioblastoma Cell Lines

**Figure 1:**
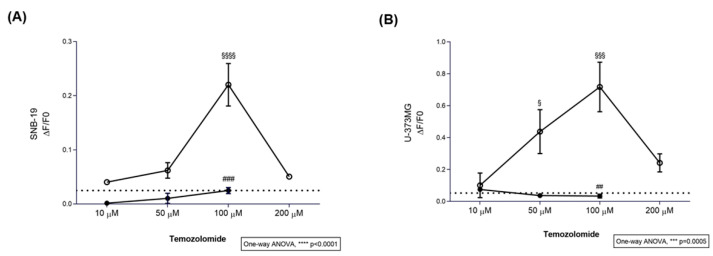
Calcium dose–response curves of (**A**) SNB-19 and (**B**) U-373MG glioblastoma cells to temozolomide. Different concentrations of temozolomide (TMZ; 10–200 μM), in the presence (●) or absence (○) of 1 mM probenecid, a known TAS2R inhibitor. Dot line: Ca^2+^ levels measured in cells with vehicle only (DMSO ≤ 0.2%). Results are presented as mean ± SEM. Statistical analysis was performed by two-way ANOVA followed by Tukey’s multiple comparisons test [N ≥ 3 independent experiments; ^§^ versus vehicle (^§^ *p* < 0.05, ^§§§^ *p* < 0.001 and ^§§§§^ *p* < 0.0001); ^#^ versus probenecid + TMZ (^##^ *p* < 0.01 and ^###^ *p* < 0.001)].

We assessed the SNB-19 and U-373MG response to TMZ (10–200 μM) using a Ca^2+^ imaging assay (Figure 1) in the presence or absence of probenecid, a blocker of TAS2R16, R38, and R43, to assess if any of these receptors could interact with TMZ eliciting a taste receptor typical response in these cells, the mobilisation of intracellular Ca^2+^ [28,29]. Our results showed that TMZ triggered Ca^2+^ mobilisation in these glioblastoma cells. TMZ at 100 µM (ΔF/F0 = 0.220 ± 0.039) triggered a significant increase in intracellular Ca^2+^ levels in SNB-19 cells (Figure 1A), compared with the vehicle (ΔF/F0 = 0.025 ± 0.008), which was abolished in the presence of probenecid (ΔF/F0 = 0.025 ± 0.006). U-373MG cells stimulated with 50 and 100 µM TMZ showed higher Ca^2+^ levels (ΔF/F0 = 0.437 ± 0.137 and ΔF/F0 = 0.718 ± 0.155) in comparison with vehicle control (ΔF/F0 = 0.052 ± 0.007) (Figure 1B). In addition, the TMZ effects on intracellular Ca^2+^ levels in the presence of probenecid were reversed (ΔF/F0 = 0.034 ± 0.012). Neither 10 µM nor 200 µM TMZ triggered significant Ca^2+^ responses in SNB-19 or U-373MG glioblastoma cells (Figure 1).

### 2.2. The Bitter Taste Signalling Pathway Modulates TMZ Cytotoxicity

**Figure 2:**
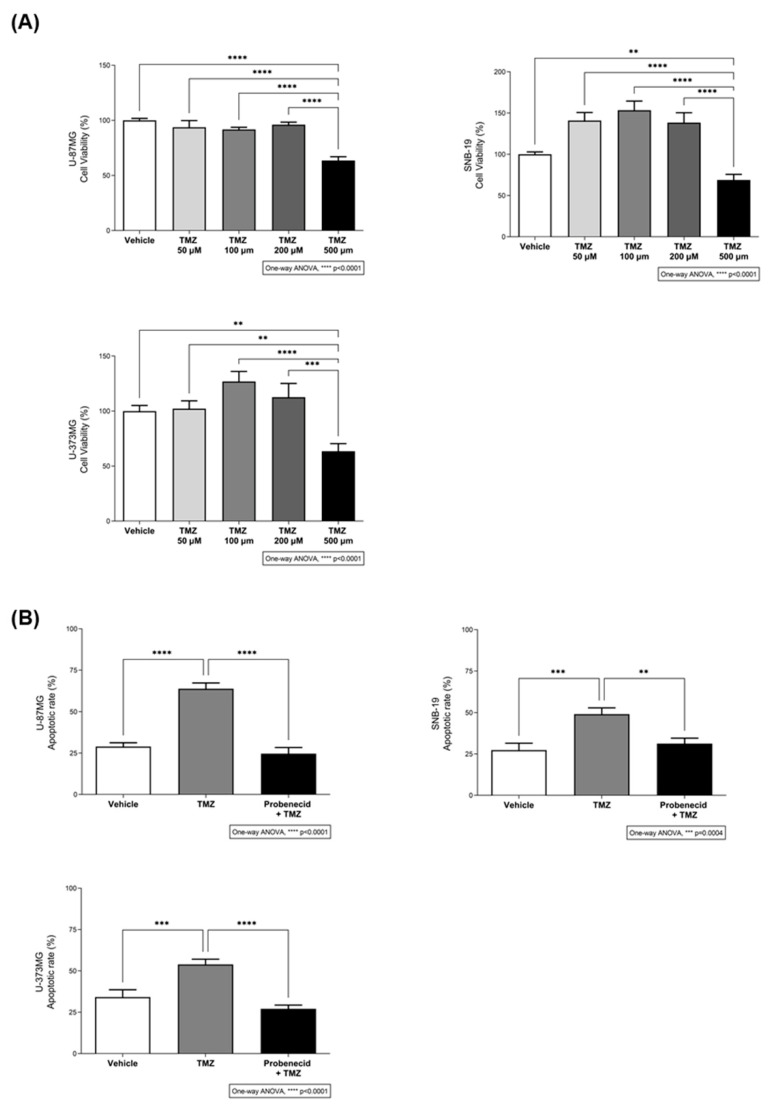
Cytotoxic effect of different concentrations of temozolomide and its modulation by bitter taste signalling. (**A**) Dose–response cytotoxicity assay of glioblastoma cells exposed to different concentrations of temozolomide (TMZ; 50–500 μM) for 72 h was assessed in U-87MG, SNB-19, and U-373MG glioblastoma cells by MTT assay. Results are presented as mean ± SEM. Statistical analysis was performed by one-way ANOVA followed by Tukey’s multiple comparisons test. [N ≥ 3 independent experiments; ** *p* < 0.01, *** *p* < 0.001 and **** *p* < 0.0001]. (**B**) The apoptotic rate of U-87MG, SNB-19, and U-373MG glioblastoma cells incubated with 500 µM TMZ for 72 h, in the presence or absence of 1 mM probenecid, was assessed by Hoechst 33342 staining. Bar graphs represent mean ± SEM. Statistical analysis was performed by one-way ANOVA followed by Tukey’s multiple comparisons test [N ≥ 3 independent experiments; ** *p* < 0.01, *** *p* < 0.001 and **** *p* < 0.0001].

Next, we proceeded to dose–response (50–500 µM) cytotoxicity assays, and found 500 µM TMZ was the minimum concentration required to reduce the viability of U-87MG (49.08% ± 1.92), SNB-19 (44.46% ± 0.97), and U-373MG (50.92% ± 0.45) cells significantly (Figure 2A).

**Figure 3:**
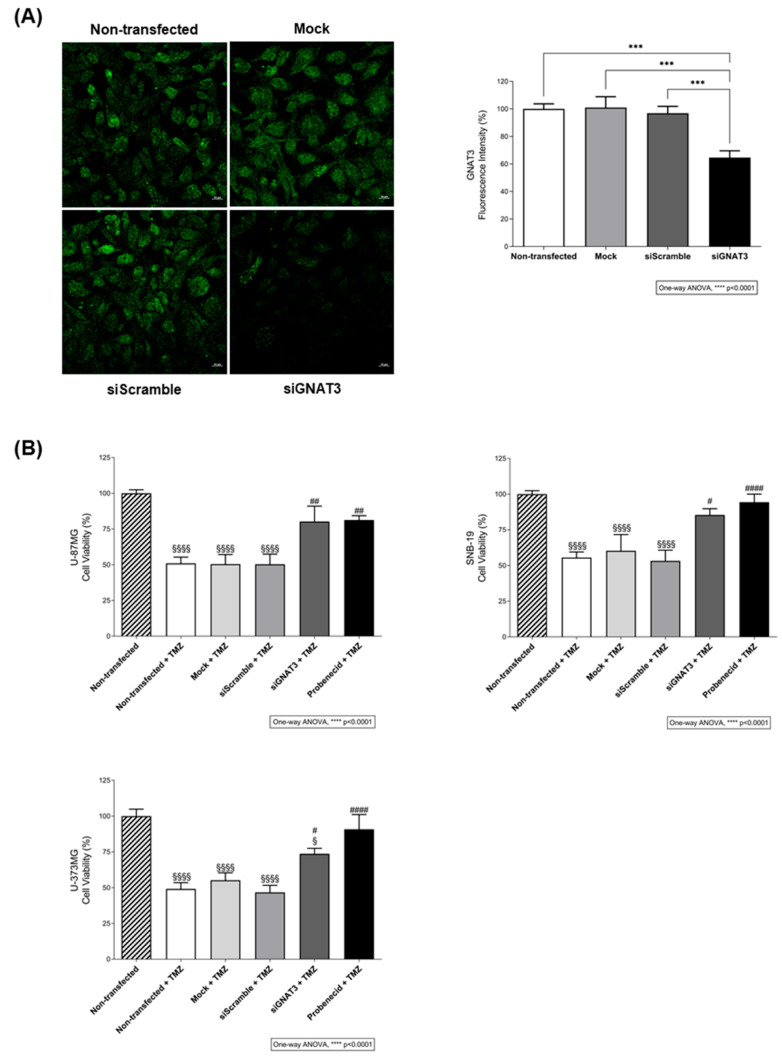
The effect of temozolomide on the viability of glioblastoma cells is attenuated by inhibition of the bitter taste signalling pathway. (**A**) Immunofluorescence analysis of α-gustducin (GNAT3) expression after siRNA transfection in SNB-19 glioblastoma cells. Protein levels of siRNA *GNAT3*-transfected cells are decreased in comparison with non-, mock-, and siRNA scramble-transfected cells. The quantification of GNAT3 fluorescence intensity (green) was performed in different regions of interest of confocal microscopy images obtained from three independent experiments. Scale bar: 10 µm. Bar graphs represent mean ± SEM. Statistical analysis was performed by one-way ANOVA followed by Tukey’s multiple comparisons test. [N = 3 independent experiments; *** *p* < 0.001]. (**B**) Effects of TMZ on the viability of U-87MG, SNB-19, and U-373MG glioblastoma cells transfected or mock-transfected for 24 h with *GNAT3* or a scramble siRNA, followed by incubation with 500 µM TMZ for 72 h, in the presence or absence of 1 mM probenecid. Bar graphs represent mean ± SEM. Statistical analysis was performed by one-way ANOVA followed by Tukey’s multiple comparisons test. [N ≥ 3 independent experiments; ^§^ versus non-transfected (^§^ *p* < 0.05 and ^§§§§^ *p* < 0.0001); ^#^ versus non-transfected + TMZ (^#^ *p* < 0.05, ^##^ *p* < 0.01 and ^####^ *p* < 0.0001)].

Then, we evaluated the effects of TMZ on glioblastoma cell apoptosis, in the presence or absence of probenecid, by staining the cell nuclei with Hoechst 33342 and counting cells with apoptotic vesicles. As expected, TMZ induced apoptosis in these cells, but this effect was reduced to control levels in the presence of probenecid (Figure 2B). In addition, TMZ-induced cytotoxicity (Figure 2A) was completely abolished by probenecid (Figure 3B), confirming the requirement for bitter taste signalling. Our observations suggested that TMZ-induced toxicity depends, at least in part, on an intact bitter taste signalling pathway. To test this hypothesis, we proceeded with knockdown experiments targeting the specific guanine nucleotide binding protein that mediates taste receptor-mediated signalling cascades, GNAT3 (Figure 3). TMZ alone induced a reduction in the cells’ viability of approximately 37.4% in U-87MG, 41.1% in SNB-19, and 45.9% in U-373MG in comparison with non-, mock- and siRNA scramble-transfected cells (Figure 3B). Notably, the presence of probenecid or the silencing of α-gustducin (GNAT3) reduced the effects of TMZ on glioblastoma cell viability (Figure 3B), reinforcing the results from the previous experiments.

Overall, TMZ elicited Ca^2+^ responses in a dose-dependent manner and showed cytotoxic effects in glioblastoma cells, which were significantly reduced in the presence of probenecid or by *GNAT3* silencing, highlighting that TMZ effects depend on a functional bitter taste signalling pathway in these cells.

### 2.3. TAS2R43 Mediates the Effects of TMZ on the Viability of Glioblastoma Cells

**Figure 4:**
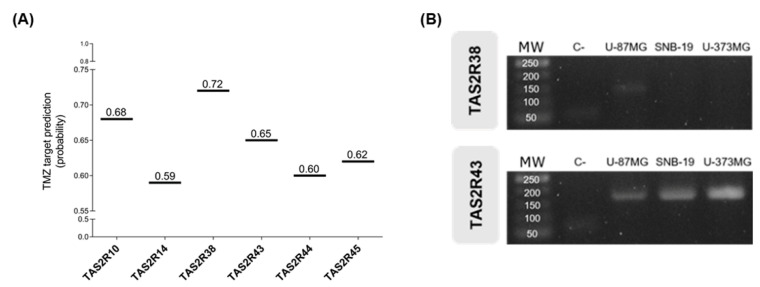
TMZ target prediction. (**A**) TMZ is predicted to bind TAS2R38 > R10 > R43 > R45 > R44 > R14 according to the webserver VirtualTaste algorithm. Results are presented as probabilities. (**B**) mRNA expression profile of *TAS2R38* and *TAS2R43* in glioblastoma cell lines (U-87MG, SNB-19, U-373MG). Only *TAS2R43* mRNA was detected in all cell lines. The identities of the amplified products were confirmed by Sanger sequencing. MW: molecular weight (base pair); C-: negative control.

After confirming that blocking the bitter taste signalling pathway reduced the cytotoxicity of TMZ in these cell lines, the next step was to identify candidate TAS2Rs contributing to this effect. Based on the webserver VirtualTaste predictions (Figure 4A), TMZ could bind six TAS2Rs: R38 (0.72) > R10 (0.68) > R43 (0.65) > R45 (0.62) > R44 (0.60) > R14 (0.59). However, only TAS2R38 and R43 bind probenecid [28,29], and the former was not expressed in all the cell lines analysed (Figure 4B).

**Figure 5:**
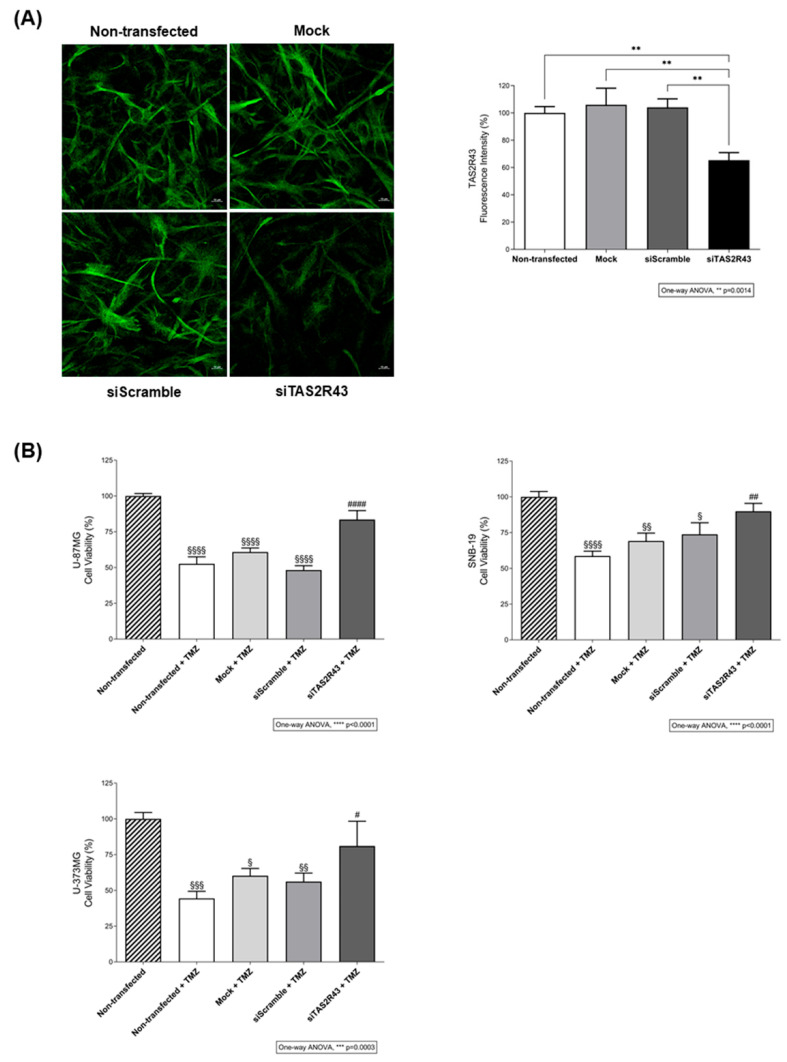
The anti-proliferative effects of temozolomide in glioblastoma cells depend on the TAS2R43 signalling. (**A**) Immunofluorescence analysis of TAS2R43 expression after siRNA transfection in SNB-19 glioblastoma cells. Protein levels of siRNA *TAS2R43*-transfected cells are decreased in comparison with non-, mock-, and siRNA scramble-transfected cells. The quantification of TAS2R43 fluorescence intensity (green) was performed across different regions of interest in confocal microscopy images from three independent experiments. Scale bar: 10 µm. Bar graphs represent mean ± SEM. Statistical analysis was performed by one-way ANOVA followed by Tukey’s multiple comparisons test. [N = 3 independent experiments; ** *p* < 0.01]. (**B**) Effects of TMZ on the viability of U-87MG, SNB-19, and U-373MG glioblastoma cells transfected or mock-transfected for 24 h with *TAS2R43* or a scramble siRNA and incubated with 500 µM TMZ for 72 h. Bar graphs represent mean ± SEM. Statistical analysis was performed by one-way ANOVA followed by Tukey’s multiple comparisons test. [N = 3 independent experiments; ^§^ versus non-transfected (^§^ *p* < 0.05, ^§§^ *p* < 0.01, ^§§§^ *p* < 0.001 and ^§§§§^ *p* < 0.0001); ^#^ versus non-transfected + TMZ (^#^ *p* < 0.05, ^##^ *p* < 0.01 and ^####^ *p* < 0.0001)].

Based on these predictions and expression data, we analysed the contribution of TAS2R43 to TMZ cytotoxicity using siRNA knockdown (Figure 5A). TMZ alone induced a reduction of approximately 41.78 ± 2.44% in the viability of U-87MG, SNB-19, and U-373MG cells in comparison with non-, mock- and siRNA scramble-transfected cells but was unable to reduce the viability of *TAS2R43*-silenced cells (Figure 5B), suggesting that TMZ-induced reduction in cell viability is TAS2R43-dependent.

### 2.4. TMZ Induces Intracellular DOX Accumulation in Glioblastoma Cells

**Figure 6:**
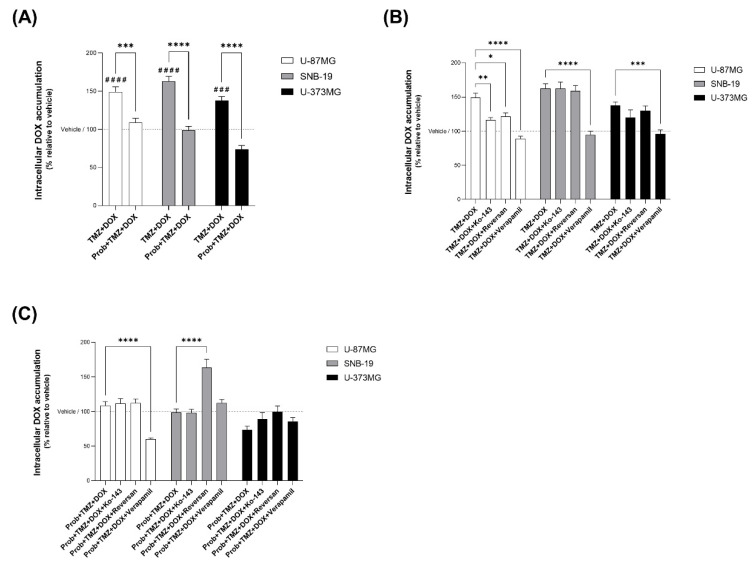
TAS2R43 signalling contributes to TMZ-induced intracellular DOX accumulation in glioblastoma cells, possibly modulation of ABC transporters. (**A**) Intracellular doxorubicin (DOX; 1 µM) accumulation in U-87MG, SNB-19, and U-373MG glioblastoma cells incubated with TMZ (1 µM) for 1 h, in the presence or absence of probenecid (1 mM), compared with vehicle (DMSO ≤ 1%). (**B**) Effects of ABCB1, ABCC1, and ABCG2 inhibition on TMZ-facilitated DOX accumulation in U-87MG, SNB-19, and U-373MG cells. Cells were pre-incubated for 1 h with verapamil (5 µM; ABCB1 inhibitor), reversan (10 µM; ABCC1 inhibitor), or Ko143 (100 nM; ABCG2 inhibitor), followed by 1 h incubation with a solution containing DOX (1 µM) and TMZ (1 µM). (**C**) Effects of ABCB1, ABCC1, and ABCG2 inhibition on DOX accumulation upon TAS2R43 inhibition with probenecid. Bar graphs represent mean ± SEM. Statistical analysis was performed by one-way ANOVA followed by Tukey’s multiple comparisons test [N ≥ 3 independent experiments; * *p* < 0.05, ** *p* < 0.01, ***^/###^ *p* < 0.001 and ****^/####^ *p* < 0.0001; ^#^ versus vehicle].

To evaluate whether TAS2R43 signalling contributes to TMZ-induced modulation of ATP-binding cassette (ABC) transporters, we assessed doxorubicin (DOX) transport, a well-known ABC transporter substrate, upon TMZ stimulation in U-87MG, SNB-19, and U-373MG glioblastoma cells. First, we showed that TMZ increased intracellular DOX accumulation by approximately 49%, 63%, and 38% in U-87MG, SNB-19, and U-373MG cells, respectively, compared with vehicle, and this increase was significantly reduced in the presence of probenecid (Figure 6A).

Next, we aimed to investigate which ABC transporter influences TMZ-facilitated DOX intracellular accumulation using specific inhibitors of ABCB1, ABCC1, and ABCG2. In U-87MG cells, inhibition of ABCB1, ABCC1, and ABCG2 significantly reduced TMZ-induced DOX accumulation, with changes in the order of 20–40%, supporting the involvement of all three transporters in intracellular DOX uptake (Figure 6B). In contrast, in SNB-19 and U-373MG cells, only ABCB1 inhibition produced significant changes in TMZ-induced DOX accumulation, whereas ABCC1 inhibition caused a 30–40% change in DOX accumulation specifically in probenecid-treated SNB-19 cells (Figure 6B). Together, these findings indicate that TAS2R43 signalling contributes to TMZ-induced intracellular DOX accumulation from ABCB1, ABCC1, and ABCG2.

Finally, the contribution of individual ABC transporters to TAS2R43-dependent DOX accumulation was evaluated by probenecid incubation. Overall, inhibition of ABCB1 or ABCG2 did not consistently modify DOX levels upon probenecid blockade, indicating that these transporters are not major downstream effectors in this context (Figure 6C). In contrast, in SNB-19 cells, ABCC1 inhibition led to a significant change (approx. 65%) in DOX accumulation in the presence of probenecid, suggesting that ABCC1 is a likely downstream effector of TAS2R43-dependent effects in mediating TMZ-induced intracellular drug accumulation in these cells (Figure 6C).

Overall, our results suggest that TAS2R43 signalling contributes to TMZ-induced intracellular DOX accumulation in glioblastoma cells, most likely modulation of ABC transporters.

## 3. Discussion

In recent years, increasing evidence has shown that several compounds, including flavonoids, alkaloids, cannabinoids, and lactones, exert anticancer effects such as anti-proliferative, pro-apoptotic, anti-angiogenic, and anti-metastatic activities in different cancers, including glioblastoma [30,31,32]. Many of these compounds are known or predicted ligands of TAS2Rs and some, such as resveratrol and epigallocatechin gallate, have been proposed as adjuvants to TMZ therapy [33].

Although TMZ is a predicted bitter-tasting compound, its potential to bind and activate specific TAS2Rs has not been addressed before. We explored whether TMZ-induced effects depend on bitter taste signalling in glioblastoma cells using functional assays with TMZ in the presence or absence of probenecid, a known TAS2R16, R38, and R43 antagonist [28,29], and by silencing the taste signalling pathway via *GNAT3* knockdown. It should be noted that TMZ concentration and exposure requirements differed between each readout. Acute Ca^2+^ imaging required relatively low TMZ concentrations to elicit rapid but non-toxic responses, whereas viability and apoptosis required higher TMZ doses and exposure. In contrast, the DOX accumulation assays used the lowest TMZ concentration (1 μM) to avoid saturating ABC efflux, allowing detection of transporter-dependent differences while maintaining cell viability.

Our findings showed that TMZ reduced cell viability, increased cell apoptosis, and elevated intracellular Ca^2+^ levels in U-87MG, SNB-19, and U-373MG glioblastoma cells. Interestingly, these TMZ effects were significantly reduced in the presence of probenecid and *GNAT3* knockdown, suggesting that bitter taste signalling pathway involvement somehow enhances the anticancer effects of TMZ. The next step was to identify TAS2R candidates to which TMZ could act as a ligand. Based on in silico analysis to predict potential target receptors for TMZ, TAS2R expression profiling in these cells, and probenecid binding, TAS2R43 emerged as the most likely TAS2R interacting with TMZ. In fact, when we silenced *TAS2R43*, the effects of TMZ in the glioblastoma cells analysed were also reduced similarly to what was seen with *GNAT3* silencing and probenecid incubation. Thus, TAS2R43 expression in these cancer cells is essential for the effective action of TMZ. Interestingly, TAS2R43 is also a target of the bitter compound epigallocatechin gallate, which in turn has been widely used as an adjuvant agent in the treatment of gliomas, especially in glioblastoma, enhancing the therapeutic efficacy of TMZ [34,35,36,37].

Because TMZ induces cytotoxicity through DNA alkylation, we hypothesised that TAS2R43 signalling might contribute to its cellular effects, potentially by modulating ABC-mediated efflux. Efflux transporters, particularly ABC transporters, remain a major hurdle in cancer therapy, limiting intracellular drug accumulation and treatment efficacy. This is particularly troublesome for brain tumours to overcome the blood–brain barrier, where efflux transporters are also very active [38,39]. Previous studies indicate that inhibition or genetic disruption of ABC transporters can potentiate the effects of TMZ and other anti-tumour drugs [40,41,42]. Clinical and experimental studies further associate the overexpression of multiple ABC transporters, including ABCB1, ABCC1, and ABCG2, with multidrug resistance and reduced TMZ responsiveness, indicating that combined transporter activity influences therapeutic outcome [18,43,44,45]. In addition, we previously demonstrated that TAS2R14 regulates efflux transport across the blood–cerebrospinal fluid barrier and modulates the disposal of bitter ligands [40]. Our intracellular DOX accumulation assays showed that TMZ increased drug retention in three glioblastoma cell lines, an effect attenuated by TAS2R43 antagonism. Transporter-specific analyses indicate that TMZ-induced DOX accumulation involves multiple ABC transporters in U-87MG cells. In contrast, in SNB-19 cells, the effect is largely ABCC1-dependent, suggesting a cell-line-specific TAS2R43–ABC regulatory relationship that may reflect distinct chemoresistance phenotypes. Increased intracellular DOX indicates reduced ABC-mediated efflux. Collectively, these results suggest that TAS2R43 signalling contributes to TMZ-induced modulation of intracellular drug availability through ABC transport in glioblastoma cells. These observations fit with previous evidence that ABC transporters at the blood–brain barrier and in glioblastoma cells can limit TMZ brain penetration and antitumour efficacy. That pharmacological or genetic inhibition of ABCB1 and ABCG2 enhances TMZ delivery to intracranial tumours and improves therapeutic response [41,46]. Furthermore, in glioblastoma, ABCC1 overexpression has been associated with temozolomide resistance, and its downregulation restores drug sensitivity [47].

In summary, we demonstrated that TMZ-induced anti-proliferative and pro-apoptotic effects in glioblastoma cells are significantly attenuated by TAS2R antagonism and TAS2R43/GNAT3 knockdown, indicating a functional role for bitter taste signalling. While these convergent pharmacological and genetic approaches strongly support TAS2R43’s role as an emerging candidate modulator of TMZ responsiveness, they do not provide direct evidence of receptor binding or activation by TMZ, and probenecid’s off-target effects were only partially mitigated by siRNA validation. Future studies will be essential to confirm TAS2R43 as a biomarker of TMZ responsiveness, to test whether pharmacological activation with selective agonists, such as epigallocatechin gallate, can further potentiate TMZ cytotoxicity, and to explore its therapeutic potential when combined with ABC transporter modulation.

## 4. Materials and Methods

### 4.1. Materials

Temozolomide (TMZ; CAS No 85622-93-1) was purchased from Cayman Chemical (#14163; Ann Arbor, MI, USA). A stock solution was prepared in dimethyl sulfoxide (DMSO) and freshly dissolved in Tyrode’s solution or culture medium before the experiments, with the final DMSO concentration not exceeding 1%. A vehicle control was included in all the experiments. Probenecid (CAS No 57-66-9), a known TAS2R16, TAS2R38, and TAS2R43 antagonist [28,29], was obtained from Sigma-Aldrich (#P8761; St. Louis, MO, USA), dissolved in 1 N NaOH at 0.17 M and diluted in Tyrode’s solution or culture medium. FURA-2AM (#F1221), pluronic acid F-127, Lipofectamine™ 2000 (#11668027), Opti-MEM medium (#11058-021), small interfering RNA (siRNA) targeting α-gustducin (GNAT3; #4392420; ID s51191; sequence: S-*GCGAGAUGCAAGAACCGUATtt* and AS-*UACGGUUCUUGCAUCUCGCtc*) and bitter taste receptor TAS2R43 (#AM16708; ID 202331; sequence: S-*CCCUACUAUCUUUUAUGCUtt* and AS-*AGCAUAAAAGAUAGUAGGGtc*), and scramble siRNA (#4390843) were purchased in ThermoFisher Scientific (Waltham, MA USA). MTT [3-(4,5-dimethylthiazol-2-yl)-2,5-diphenyltetrazolium bromide] was purchased from Gerbu Biotechnik GmbH (#1006; Heidelberg, Germany).

### 4.2. Cell Culture

Human malignant glioblastoma cell lines U-87MG and U-373MG were kindly provided by Dr. Joseph Costello (University of California, San Francisco), and SNB-19 was obtained from the German Collection of Microorganisms and Cell Cultures (Braunschweig, Germany). These glioblastoma cell lines have different morphologies and proliferation rates: U-87MG are cluster-invasion cells, whereas the most chemoresistant SNB-19 and U-373MG (similar to U-251MG) are individual-invasion and expansive-growth cells, respectively [48,49].

All cell lines were grown in Dulbecco’s modified Eagle’s medium (DMEM) with high glucose and stable glutamine (bioWest, Nuaillé, France: #L0103), supplemented with 10% (*v*/*v*) FBS and penicillin (100 IU/mL)/streptomycin (100 μg/mL), and incubated in a humidified atmosphere containing 5% CO_2_ at 37 °C.

### 4.3. Evaluation of the Impact of TAS2R on TMZ Cytotoxicity

#### 4.3.1. Effects of TMZ in Intracellular Ca^2+^ Responses of Glioblastoma Cells

Intracellular Ca^2+^ mobilisation is a marker of G-protein-coupled receptor activation, including taste receptors. Thus, we measured intracellular Ca^2+^ oscillations in response to TMZ stimulus by single-cell Ca^2+^ imaging assays, in the presence or absence of the TAS2R antagonist probenecid, in glioblastoma SNB-19 and U-373MG cells. Briefly, approximately 3.5 × 10^4^ glioblastoma cells were seeded in μ-slide 8-well ibiTreat (Ibidi, Gräfelfing, Germany; #80826) and grown to 60–70% confluency, followed by measurement of changes in intracellular Ca^2+^ levels after stimulation. Glioblastoma cells were loaded with 5 μM FURA-2 AM and 0.02% Pluronic acid F-127 in culture medium for 45 min. Next, cells were washed twice with Tyrode’s solution pH 7.4 [NaCl 140 mM, KCl 5 mM, MgCl_2_ 1.0 mM, CaCl_2_ 2.0 mM, Na-pyruvate 10 mM, Glucose 10 mM, HEPES 10 mM, NaHCO_3_ 5.0 mM] and loaded with Tyrode’s for 30 min. After that, dose–response experiments were performed with a range of TMZ concentrations (50–200 µM), in the presence or absence of 30 min incubation with probenecid (1 mM). Because 200 µM TMZ failed to elicit robust Ca^2+^ elevations and could adversely affect cell viability during imaging, this condition was not carried out in the presence of probenecid. The μ-slide plates were placed on a Widefield Axio Observer Z1 inverted microscope (Zeiss, Oberkochen, Germany). Stock solution of TMZ and probenecid was freshly prepared in Tyrode’s solution before the experiments. The stimulus was applied manually with a micropipette after the baseline was recorded. The intracellular Ca^2+^ levels were evaluated by quantifying the ratio of the fluorescence emitted at 520 nm following alternate excitation at 340 nm and 380 nm, using a Lambda DG4 apparatus (Sutter Instrument, Novato, CA, USA) and a 520 nm bandpass filter (Zeiss) under a Fluar 40x/1.30 Oil M27 objective (Zeiss). Data were processed using the Fiji software v1.54r (MediaWiki, San Francisco, CA, USA). Changes in the fluorescence ratio (F = F340/F380) were measured in at least 20 cells across three or more independent experiments. Response intensity, or intracellular Ca^2+^ variation (ΔF/F0), was calculated in the following way: ΔF/F0 = (F − F0)/F0, where F0 corresponds to fluorescence ratio average at baseline (2 min acquisition before stimulus) and F corresponds to maximum peak of fluorescence ratio evoked by the stimulus applied to the cells.

#### 4.3.2. Evaluation of the Impact of GNAT3 Knockdown on TMZ Cytotoxicity

After Ca^2+^ imaging experiments, the cytotoxicity of TMZ was assessed in glioblastoma cells. Initially, a range of TMZ concentrations (50–500 μM) were used, and significant effects on cell viability were observed only at 500 μM. Thus, MTT experiments with probenecid and GNAT3 knockdown were carried out at this TMZ concentration to further support the notion that specific activation of the bitter taste signalling is elicited by TMZ. The apoptotic effect of TMZ, in the presence or absence of probenecid, was also evaluated in glioblastoma cells by Hoechst 33342 nuclei staining. Apoptotic nuclei were identified based on classical morphological criteria (chromatin condensation and nuclear fragmentation), as widely used in cancer models [50]. Glioblastoma cells were seeded on a coverslip, and stimuli were applied by incubating the cells for 72 h with TMZ (500 μM) or vehicle (DMSO 1%), in the presence or absence of 1 mM probenecid diluted in culture medium. After removing the medium, cells were fixed with PFA 4% for 10 min followed by incubation for 10 min with Hoechst 33342 (diluted 1:1000). After several washes, cells were mounted onto microscope slides and visualised under a confocal microscope LSM 710 (Zeiss) using a magnification of 63x (Plan-Apochromat 63x/1.4 Oil DIC M27). Apoptotic cells were distinguished from healthy or necrotic cells by the observation of condensed DNA and fragmented nuclei. The apoptotic rate was calculated as the ratio of apoptotic cells to total cells.

For *GNAT3* knockdown using a specific siRNA, cells were grown to 60% confluency or transfected for 24 h with a mixture of *GNAT3* siRNA (10 nM) and Lipofectamine™ 2000 in Opti-MEM medium, following the manufacturer’s instructions. A scramble siRNA (10 nM) was also used as negative control for *GNAT3*-specific targeting. Then, cells were incubated for 72 h with TMZ (500 μM) or vehicle (DMSO 1%), in the presence or absence of 1 mM probenecid diluted in culture medium. Then, 100 μL culture medium was removed and 10 μL of MTT solution (5 mg/mL in PBS) was added for approximately 45 min at 37 °C in a humidified atmosphere containing 5% CO_2_. Untreated and ethanol (70%)-treated cells were used as negative and positive controls, respectively. Following MTT incubation, formazan crystals were dissolved in DMSO for 15 min, and absorbance was read at 570 nm in a microplate spectrophotometer xMark™ (Bio-Rad, Hercules, CA, USA). Glioblastoma cell viability was expressed as a percentage of the absorbance determined in the vehicle control.

### 4.4. Identification of the TMZ Target TAS2R on Glioblastoma Cells

Following the demonstration that the bitter taste signalling pathway was involved in the mediation of the effects of TMZ, we assessed in silico which TAS2R could have a higher likelihood of binding to TMZ according to the webserver VirtualTaste method [51]. Using this approach, six TAS2Rs—TAS2R10, R14, R38, R43, R44 and R45—were identified as possible target receptors for TMZ. *TAS2R38* and *TAS2R43* were both detected in glioblastoma cells (U-87MG, SNB-19, and U-373MG) by RT-PCR [52]. Additionally, TAS2R43 protein detection in glioblastoma cells was assessed by immunocytochemistry, as described before [52]. Finally, the role of TAS2R43 in the response to TMZ was assessed in glioblastoma cells after *TAS2R43* knockdown using a specific siRNA, as described in Section 4.3.2 for *GNAT3*.

### 4.5. Effect of TMZ on ABC Transporters

Since TMZ is highly lipophilic and triggers cell apoptosis through DNA methylation and damage, we explored the hypothesis that the reduced effects of TMZ observed upon TAS2R43 knockdown could result from reduced cellular drug concentrations. Thus, we tested whether TAS2R43-mediated downregulation of ATP-binding cassette (ABC) efflux transporters, such as ABCB1, ABCC1, and ABCG2 [53]. These efflux transporters are expressed in glioblastoma and have been implicated in multidrug resistance and restricted brain penetration of anticancer drugs [38,43,44,54]. Thus, TMZ-induced intracellular accumulation of doxorubicin (DOX), a well-known substrate of these efflux transporters, was evaluated in U-87MG, SNB-19, and U-373MG glioblastoma cells. In these assays, DOX was chosen as a fluorescent probe, allowing functional assessment of their activity in a single experimental setup. Reference inhibitors of ABCB1 (verapamil), ABCC1 (reversan), and ABCG2 (Ko143) were used to dissect the contribution of each transporter to TMZ-induced changes in DOX intracellular accumulation [55,56,57].

Cells were seeded in multi-well plates and maintained under standard culture conditions until use. On the assay day, cells were washed and pre-incubated for 1 h with verapamil (5 µM), reversan (10 µM), or Ko143 (100 nM) in the presence or absence of probenecid (1 mM). After this pre-incubation period, cells were incubated for 1 h with a solution containing DOX (1 µM) and TMZ (1 µM) to allow intracellular accumulation under the different conditions. At the end of the incubation, cells were washed with ice-cold PBS, then lysed with Triton X-100 1% in PBS for 30 min at 37 °C, protected from light. The fluorescence of DOX in the lysates was quantified using a SpectraMax Gemini spectrofluorometer (Molecular Devices, San Jose, CA, USA) at 480/508 nm. In each plate, wells containing only DOX/TMZ solution were included as positive controls for the fluorometric detection of each compound. Data were expressed as relative intracellular accumulation compared with the corresponding vehicle-treated controls (DMSO ≤ 1%).

### 4.6. Data Analysis

Statistical analysis and dataset comparisons were performed using GraphPad Prism 9.3.1 (GraphPad Software). Statistical significance was determined by One-Way ANOVA followed by the software’s recommended multiple comparisons post hoc test. Results are presented as mean ± SEM of at least three independent experiments, and data were considered statistically different for a *p*-value < 0.05.

## Data Availability

The original contributions presented in this study are included in the article. Further inquiries can be directed to the corresponding author(s).
